# Memoirs of the Royal Academy of Medicine, Volume the Second

**Published:** 1833-04-01

**Authors:** 


### XIII.

MÉMOIRES DE L'ACADÉMIE ROYALE DE MEDECINE. Tome Deuxieme. 1er et 2e Fascicules. Avec Planches. Baillière, Paris et Londres, 1833.

WE are indebted to M. Baillière for this and for many other valuable French works, which we propose to notice. We propose, in the subsequent numbers of this Journal, to dedicate more space than we have lately done to continental medical literature. We are induced to do this for two good reasons:—first, because it contains very much that is valuable, and secondly, because what it contains is inefficiently disseminated in this country. Our relations with France are becoming every day more close, and it is our mutual interest that it should be so. This is not more true politically than scientifically, and whilst we are as anxious as any to uphold our national reputation, we must candidly avow that France can boast of a body of scientific men, which would shed a lustre on the brightest era of civilized nations. Her institutions are, we speak advisedly, preferable to our own—they breathe a more liberal spirit, and encourage a more generous emulation. We are too much a people of shopkeepers.

We shall take all possible opportunities of laying before our readers the substance of the best French works that appear, and we venture to hope that our plan will not be unproductive of benefit. We cannot carry it into full operation in the present number, but shall merely content ourselves with a glance at one or two recent publications.

That now before us is the production of the Royal Academy of Medicine. It contains an elaborate paper by M. Breschet, which we will notice more at length bye-and-bye, and memoirs from the pen of MM. Lisfranc, Ricord, Duchesne, and other gentlemen. The paper to which we will first direct attention is by M. Ricord, surgeon to the Hôpital des Vénériens, and purports to be founded on some facts which he has observed in that institution. We shall proceed to the consideration of the paper with as little preface as possible.

It would be impertinent in us to tell our readers that the venereal disease is surrounded with difficulties; they well know that. But it might appear more startling to some were we to assert that the late investigations on the non-administration of mercury have been productive of much general delusion. Yet, startling as it may appear, such we believe to be the fact. Yes, we believe it to be the fact, that much delusion exists with respect to the practice of treating the venereal without the aid of mercury, and much as the experiments of the army surgeons have been lauded, and great as has been the benefit accruing from those experiments, we think they have been too implicitly trusted to, or too narrowly reasoned on. This may sound to many like a heresy: we will not at present attempt to defend ourselves, but we mention our opinion, not lightly taken up, as an evidence that the investigation is not exhausted, and that much is necessary to produce general conviction on any essential point. Hereafter we may enter more in detail, and develop more particularly such ideas as we may be led to entertain upon the subject. We must confess that we think there is an opening for a really good and practical work on this disease. But to proceed to notice the facts and opinions of M. Ricord.

Of one hundred female patients in the venereal hospital, sixty, according to M. Ricord's computation, are affected with discharges acute or chronic, the blennorrhœa being always more common than the blennorrhagias. It is well known that women of the town are nearly always anxious to leave the hospitals as soon as possible, in order to return to their profession. As soon as the more prominent symptoms have disappeared, and when they are apparently approaching to a cure, they frequently elope. This is sufficiently common at the Lock Hospital in London. In Paris where, in order to follow their trade, they are under the necessity of possessing a certificate of health, it becomes an important matter to deceive their surgeons into granting it at as early a period as possible. The charge of the public health, on the other hand, being thus confided so especially to the surgeons, it becomes their duty to avoid intentional imposition and unintentional error. Now it must strike very many, as it certainly has struck us, that whilst venereal sores are extremely frequent in men, they are comparatively rare in women. Of the tenants of the Lock Hospital the majority of males have or have had sores, whilst the great majority of females have discharges. And yet those females being of the lowest class of prostitutes would probably be, and no doubt are the chief means of propagating sores. How are we to account for this anomaly? Are we to suppose that women having no sores will occasion them in men—that there exists therefore a considerable difference between the characters of the disease in the two sexes—or that sores do exist, but escape our notice. We shall presently shew that M. Ricord has in part explained this mystery, by shewing that ulcerations are not unfrequent in the deeper parts of the vagina, those parts where surgeons never search for them. But we doubt whether this is the whole of the truth, and we think, for reasons which we need not at present bring forward, that women *are* less subject to primary sores than men, and that males will contract sores followed, or liable to be followed, by secondary symptoms, from women that have merely discharges. To return to M. Ricord.

This gentleman being convinced of the inefficiency of the usual method of examination, determined never to discharge a patient without inspecting the interior of the vagina and neck of the uterus by means of the speculum. The employment of this instrument has subsequently been enforced in every instance, and, since it has been so, M. Ricord has been amply satisfied how inexact the common mode of exploration is. He entertains no doubt that the usual inspections of the public women of Paris are perfectly illusory, and offer no security. The fact has been already noticed by our own army surgeons. One gentleman, we at this moment forget who, but we think it is Dr. Hennen, remarks that he was frequently astonished at the results of the examinations of the women at Valenciennes. They were found to have merely discharges, yet sores of all descriptions were frequent among the soldiers who cohabited with them.

*Acute Blennorrhagia.* It is commonly known that the whole genito-urinary mucous membrane in the female may be the seat of gonorrhœal discharge, but it is not commonly supposed that the urethra is much affected. M. Ricord, on the contrary, has found pus issue from that canal in eight cases out of twelve. The shortness of the canal, its position, and the frequency of micturition, frequently prevent this fact from being discovered. But if a proper time be selected, and the finger introduced into the vagina, so as to compress the urethra from behind forwards, M. Ricord maintains that the fact will be found to be as he states it. All the females who had urethritis as well as vaginitis attributed their complaint to infection. M. Ricord has found acute buboes coexist with urethro vaginal blennorrhagia; —he has not found this to be the case when the vagina only was affected.

The following are the results of examination with the speculum in the state of acute blennorrhagia.

1. The mucous membrane merely unnaturally red throughout its whole extent.

2. In some patients this, attended with much heat, sensibility, and some tumefaction, has terminated without giving rise to discharge; in the majority of cases it has been succeeded by discharge.

3. In some women there were prominent patches, of various size, the redness of which was decided and terminated abruptly, whilst the remainder of the vagina was natural.

4. In some the vaginal mucous membrane presented a great number of reddish papulæ; in others it was merely spotted.

5. These different states coincided with vaginal secretions of different kinds: in one instance mucous and transparent, in another serous and reddish, in a third purulent.

6. In some of those with whom the secretion was reddish it sometimes became bloody; the reddest parts were then deficient in epithelium, and there was erosion of the mucous membrane; most frequently, however, these erosions gave rise to a purulent secretion.

7. One patient of very lymphatic temperament, affected with acute vaginitis, had the vagina studded with granulations, resembling the luxurious ones that form in scrofulous sores; in this case there was a profuse purulent discharge. M. Ricord has found a less degree of the same granular state, depending apparently on enlargement of the inflamed mucous follicles, in several patients affected with purulent discharges.

8. Three patients affected with recent purulent discharges, and sent to the hospital for gonorrhœa, had ulcerations of the vagina from three to six lines in diameter. The ulcerations were slightly infundibuliform, with excavated edges (bordés à pic) and greyish base. There was no ulceration of the external parts.

9. The different conditions of the vagina already mentioned were met with on the mucous membrane of the neck of the uterus, which in a number of cases appeared alone affected. Sometimes that portion of the mucous membrane covering the os tincæ was very evidently affected.

10. Along with the inflammation of the mucous membrane, the neck was often found enlarged, and in some cases the body of the uterus appeared slightly tumefied; the secretions of the mucous membrane of the neck were like those of the vagina.

11. In many patients presenting on their admission puriform matter at the orifice of the vagina, unaccompanied by symptoms of inflammation in the internal genital parts, and in whom the discharge had only lasted a few days, the vagina has been found healthy throughout its whole extent, whilst the os tincæ, tumefied and red round its orifice, allowed a great quantity of puriform mucus to escape from its cavity, or from that of the body of the uterus.

12. M. Ricord has observed, in upwards of a hundred cases, the following circumstance. The mucous or muco-purulent uterine secretions so frequently met with in company with other affections of the vagina, or the neck of the uterus, and frequently called "whites," have always a glairy consistence, like that of white of egg; those of the vagina, on the contrary, have not that consistence.

13. In the acute stage, M. Ricord has found that the ulcerations of the neck of the uterus are situated nineteen times out of twenty at the orifice, and once in twenty on the circumference of the neck, more or less contiguous to the cul-de-sac, formed by the vagina with the cervix. Out of six cases of the latter kind, the ulceration in four was situated on the anterior surface, and in two on the posterior. This is accounted for by the fact that in prostitutes the uterus is commonly anteverted, so that the superior face of the neck looks forwards, and is therefore most directly exposed to infection.

14. The ulcers of the neck, whatever have been their precise situation, were eighteen times out of twenty of the elevated kind—the *ulcus elevatum*. Often they were a sort of granulations and mucous tubercles; frequently they resembled granulations in groups; and in one patient there were true pustules with a white apex. In some there was erosion of the mucous membrane, such as has been adverted to as existing in the vagina. In others, and M. Ricord possesses six cases of this description, the ulcerations near the orifice, stretching into the cavity, or placed on the circumference of the neck presented all the characters attributed to the true syphilitic chancre. It should be mentioned again that in none of these patients were there any external symptoms, nor any internal ones calculated to raise suspicions as to the existence of the lesions enumerated.

M. Ricord next describes the appearances observed in chronic discharges.

1. The posterior part of the vagina is more affected than the anterior, the reverse being commonly the case in the acute form.

2. In some patients the mucous membrane is whitish, almost granular, and furnishes a lactiform secretion staining the linen white. In some this secretion becomes caseous, as it were, on coming in contact with the air, or on drying. When viewed around the neck of the uterus it greatly resembles the sebaceous secretion between the male glans and prepuce; this state may be considered as that of leucorrhœa.

3. The vaginal mucous membrane may secrete a serous fluid, staining the linen white, and having sometimes a red zone.

4. Most commonly the discharges in blennorrhœa were purulent, yellow, or greenish. The mucous membrane was then sometimes pale, at others thickened in different parts, appearing as if deprived of epithelium, or often reddened and softened, and looking like the conjunctiva in chronic ophthalmia.

5. Two patients presented in the posterior and deepest part of the vagina patches much redder than the remainder of the mucous membrane, bleeding easily, and giving rise to a purulent secretion. These patches were superficially ulcerated, and led M. R. to believe, prior to his examination with the speculum, that there was a slight loss of the substance of the uterus.

6. Along with the blennorrhagia M. Ricord found granular, pedicular, and cauliflower vegetations throughout the whole extent of the vagina, and on all the points of the vaginal region of the uterus. Sometimes the vagina alone was affected, sometimes only the neck of the uterus. In some of the patients nothing externally announced with any certainty the existence of these vegetations within, a more or less puriform chronic discharge being the only symptom. In one there existed at the base of a vegetation seated two inches behind the carunculæ myrtiformes, a deep, elongated, irregular ulceration, appearing to have been the result of a laceration.

7. Catarrh of the uterus was found amongst the greater number of patients. The discharge from the os uteri was sometimes transparent, with the cervix pale; sometimes more opaque, with redness of the cervix, or even slight ulcerations of the orifice; sometimes purulent, with the cervix red and thickened. There existed also erosions round the os uteri, or even true ulcers, elevated or deep.

8. In one patient who had had many children, and in whom the cervix was much developed, with a very large orifice, nothing was seen externally; but on separating the lips of the os tincæ, by means of the speculum, there were seen on their internal surface ulcerations which extended into the uterine cavity.

9. These various ulcerations appeared to M. Ricord the most common cause of those interminable discharges, which, though termed leucorrhœal, are not the less contagious.

10. Finally, in some females muco-purulent discharge issuing from the uterine orifice was the only appreciable symptom.

M. Ricord discovered on the cadaveric examination of two patients, who died in the hospital, the following facts. In one female, who was thought to be affected with blennorrhagia only, there was found a deep infundibuliform ulceration, with a hard base, situated an inch and a half behind the carunculæ myrtiformes. In the other were two ulcerations, having a character similar to that of the preceding, but irregularly elongated in form, one upon the anterior lip of the os tincæ, the other extending into its cavity; the last was a little rounded.

M. Ricord mentions one or two facts on the subject of contagion.

A woman of the town, who had been under treatment upwards of a month for an *elevated ulcer* of small extent on the left side of the os tincæ, with slightly purulent uterine catarrh, but without any decided vaginal discharge, was examined with the speculum on the day of her dismissal. The only thing which was observed was the circumstance that the ulceration of the orifice was not completely healed, a point, about the size of a large pin's head remaining, but apparently on the point of being cicatrized. The patient was dismissed. Immediately after her dismissal a pupil of M. Ricord's, who had not had connexion for some time previously, had intercourse with her. He contracted an elevated ulcer at the base of the gland, and a bubo. The patient returned to the hospital two days after her dismissal. On examination with the speculum, nothing was observed in the external parts, but the neck of the uterus was red, a little swollen, the cicatrix of the ulceration broken, and this doubled in size and secreting a puriform discharge. The patient was kept in the hospital until she was perfectly cured.

A woman, just admitted, and affected with ulceration of the os uteri, deep but of small extent, and giving rise to a purulent discharge, unattended with any external ulceration, said that her husband had a chancre. This man was also in the hospital; there was a chancre at the meatus.

Three patients, two in the civil and one in the police ward, affected with purulent discharge and red ulcerated granulations of the os uteri, told M. Ricord that every time they had connexion with men, they communicated to them a violent gonorrhœa, but never chancre.

The following is the resumé of the facts observed by M. Ricord.

1. That the vulva is more frequently affected in blennorrhagia than in blennorrhœa.

2. That in chronic discharges, on the contrary, the deep parts of the vagina, the neck of the uterus, and its cavity, are more frequently affected.

3. That the different ulcerations are most frequent in the parts of the vulva, situated anterior to the carunculæ myrtiformes, then on the neck of the uterus, and lastly in the deep parts of the vagina.

4. That vegetations are met with in the following order of frequency—vulva—vagina—uterus.

5. That the different lesions in the acute stage may exist consentaneously on different points.

6. That acute affections may be associated with chronic affections that pre-existed, and that they may be speedily cured, without the others being influenced.

7. That on the contrary the chronic affection commonly becomes more severe in consequence.

8. That the various lesions tend to produce similar ones by contagion, and that we must not admit that a severe gonorrhœa in a female can produce chancres in the male.

9. Finally, that a woman affected with gonorrhœa and chancres, and having connexion with several men, may communicate chancre only, or gonorrhœa only, or both at once.

We must say that we consider the foregoing facts extremely important, as important perhaps as any that have been elicited in modern times, with reference to the venereal disease. They add to our amount of exact knowledge, and every addition of this kind is a great gain. We hope we shall never again see such a work as Hunter's, a work unphilosophical in its conception and absurd in its conclusion, a work that never did good and never can do any. Based on an assumption purely gratuitous, it has led to ideas unnaturally restricted, and to practice mischievously empiric. Setting out with the notion that syphilis has one peculiar form, and that the sore which he denominatad chancre is that form, he has forced his disciples to come to the conclusion that syphilis has nearly deserted the earth, and that chancre has passed away with the Hunter's. Certain it is that such a sore is now comparatively rare, and that five or six cases of other sores are witnessed for one of it. But to return to our subject.

We have said that the investigations of M. Ricord have added to our amount of exact knowledge, and we need hardly say that without exact knowledge there is no true philosophy. They have contributed exactness to a subject in which it was particularly wanted, that of the female discharges. How often must surgeons have seen such a case as this. A gentleman presents himself with a primary sore, and it is necessary, or at all events judicious, to give him mercury. The female with whom alone he has been connected is examined, and merely a little discharge is discovered. The point seems difficult, but like all familiar difficulties, the surgeon gets over it without his suspicions being much excited or his faith in common opinions much shaken. He looks upon it as one of the difficulties of the venereal, and puts up with it.

All must be struck with the fact, that in men sores predominate, in women discharges. Nay, more than this. A woman has distinct secondary symptoms independent of the exhibition of a grain of mercury. Yet merely a history of primary discharge is obtained. Here it is commonly supposed that the woman errs from inaccurate observation, or that she intentionally deceives. But women will be admitted into a Lock Hospital with discharge, there will be nothing else discoverable, and secondary symptoms, eruptions, will occur under the surgeon's observation. Such facts are always blinked, for no man, and particularly no prejudiced man, will think much about what he does not wish to doubt.

The investigations of M. Ricord prove that we have laid up a false experience, that we know little, because our knowledge has been inexact. We must begin again. There is no disputing it. Every surgeon must use the speculum, at least we must use it where we can, and at no hospital should it be neglected. It is proved that ulcerations of the deep parts of the vagina and of the cervix and os uteri do frequently exist, when nothing but discharge is visible externally. That fact being known, what candid man would in future trust to others or himself, if means of arriving at the whole truth had not been resorted to in the investigation of the organs of the female? It is idle to say, that the use of the speculum is not new. Its general use, that is, its use in *all* cases is new, and that is the important point. In fact, the whole matter lies in a nut-shell, and none can mistake it. We have hitherto been treating as discharges only, what may have been deep ulcerations, or other lesions, accompanied with discharges. The doubt is sufficient to vitiate all conclusions drawn or to be drawn from such a source.

M. Ricord does not appear to us to have communicated the result of his examinations in a clear or satisfactory manner. He has given us no data of comparison, no means of ascertaining the frequency of such or such vaginal or uterine lesion in a given number of cases. The manner in which his propositions are stated is more brief than lucid. However, these are minor considerations, and we cannot but thank M. Ricord, as no doubt the profession will do, for putting the important facts, to which we have adverted, in a prominent point of view. We suppose that their having occurred at a public hospital, together with the circumstance of their being brought forward in the Memoirs of the Academy of Medicine, will be a sufficient guarantee for their general accuracy.

We have now to notice the observations of M. Ricord on the treatment of the disease. On this we shall be more brief.

In the acute stage M. Ricord recommends antiphlogistic treatment, and he seems to approve of general bleeding, practised and recommended by Lisfranc. If leeches are employed it should be above Poupart's ligament, or, if the anus is affected, over the scrotum, as otherwise the discharge reaching the leech-bites, occasions very troublesome sores. M. Ricord prefers general to hip-baths. He thinks injections often injurious from the irritation occasioned by the introduction of the pipe of the syringe, and applies charpie dipped in an emollient decoction and often renewed. As soon as the acute symptoms have subsided the speculum should be introduced in order to ascertain the exact condition of the parts.

He has almost entirely abandoned injections. Patients too commonly use them carelessly, and even when used carefully, they are too frequently unsuccessful. In a great number of cases the injection never reaches the os uteri. M. Parent du Chatelet ascertained this fact, by placing on the os tincæ a plug of lint, and ordering an injection of coloured fluid to be used; the lint was not stained by the injection. Injections may be made to reach the deepest parts of the vagina, by a proper inclination of the pelvis, but they remain but a very short time in contact with the deeper parts. In short, M. Ricord prefers the introduction of dossils of lint soaked with the fluid to be employed, retaining them from twelve to twenty-four hours according to the circumstances of the case. If the discharge is slight a dossil of charpie dipped in a concentrated solution of the acetate of lead is introduced deeply into the vagina by means of the speculum, and replaced every twenty-four hours. If the discharge is profuse, the lint is renewed twice daily. Many patients were rapidly cured in this manner, who had been employing astringent injections of all kinds ineffectually.

If the mucous membrane is covered with prominences like pale granulations, or softened, the introduction of a dossil of charpie soaked in a solution of one part of acid nitrate of mercury in twelve of water, and left according to the state of the parts and irritability of the patient, ten minutes, a quarter of an hour, half an hour, or more, and then followed by a similar application of pure water, has succeeded in many instances.

Ulcerations of the vagina or uterus are often healed by the mere application of cold water. If they are accompanied with swelling of the parts where they are situated, small abstractions of blood are useful; if languid, M. Ricord cauterises them with the acid nitrate of mercury applied by dressing forceps, and immediately introduces a dossil of lint soaked in a solution of acetate of lead, and renewed every twenty-four hours or oftener.

Ulcerations of the cervix uteri being frequently accompanied with uterine discharge, the mucus covers the ulcers. The mucus should be cleared away with lint, or removed by coagulating it with the nitrate of mercury prior to the cauterization of the ulcers themselves. M. Ricord has cauterized ulcerations of the cervix in upwards of sixty patients without experiencing the slightest accident. The cauterizations have been repeated every six or eight days, avoiding the catamenial period, and it has never been repeated more than ten times. Females have been thus cured who seemed incurable.

Transparent uterine discharges have often disappeared or have been much diminished, when the ulcerations on the cervix have been healed. Some opaque uterine discharges have subsided in the same manner. In five cases purulent discharges existed alone, and appearing to be kept up by atonic ulcerations within the cavity of the cervix, injections of the nitrate of mercury were thrown into the cavity. Three of these discharges were radically cured; the remaining two became transparent and less abundant. M. Ricord employs for injecting the uterus a double syringe with a double pipe. One part of the syringe contains a solution of the nitrate of mercury, the other water. An elastic tube, open at its two ends, is adapted to the double pipe. One end of the catheter is introduced into the cavity of the cervix, into which a small coffee-cup full of the solution of the nitrate is injected, and after being allowed to remain for an instant, is driven out by the water. All the patients who have submitted to these injections have experienced immediately afterwards pains in the loins, and a sensation of heat in the hypogastrium; these symptoms disappear after the hip-bath. Two patients had five injections thrown up, the others three or four. They were employed at intervals of eight days. The solution of the acetate of lead succeeded in several cases of transparent uterine catarrh.

We conclude by again directing our readers' attention to this Memoir. We do not care for the treatment nor even for the particular facts, so much as for the principle involved—that we cannot pronounce on the source of a vaginal discharge with any certainty, unless we are fully acquainted with the state of the deeper parts.

